# Supplementary material for: Seqwin: ultrafast identification of signature sequences in microbial genomes
Source: Bioinformatics. 2026 Jul 7;42(Suppl 1):btag261. doi: 10.1093/bioinformatics/btag261 (PMC13340262; doi:10.1093/bioinformatics/btag261)
Supplement: btag261_Supplementary_Data [file Response Letter.pdf › Response Letter.pdf]

SUBMISSION: 402
TITLE: Seqwin: Ultrafast identification of signature sequences in microbial genomes

------------------------ METAREVIEW ------------------------
This manuscript presents a clear problem (detection of microbial genomic signatures) that is of value to microbiologists, and then provide a working solution. The manuscript is well-written, the technical details check out, and the benchmarking seems broadly reasonable.

The reviewers highlight a few missing pieces that would make the manuscript even stronger, including some more experiments characterizing the nature of the signatures generated, and how they compare to other tools. There are also a few minor presentation concerns that ought to be addressed before publication.

Having said that, the manuscript is suitable for acceptance to ISMB.

The authors thank the chairs and the reviewers for their highly valuable feedback and comments.

In addition to addressing the comments from the reviewers, we also updated Seqwin benchmarks using the latest version (same output signatures but with reduced running time). We also uploaded benchmarking datasets, outputs and scripts to Zenodo.

---------------------- REVIEW 1 ---------------------

SUBMISSION: 402
TITLE: Seqwin: Ultrafast identification of signature sequences in microbial genomes

----------- Overall evaluation -----------
The authors of this method have identified a good problem to solve, and use a clever and modern approach to do so. The results, both in terms of computational efficiency and selection of likely good-quality probes are good. These results are clearly a big advance over the tools the authors compare their approach to.

Two main pieces are missing in my view:
- The number of non-target genomes used is relatively small. Restricting to neighbouring taxa is reasonable, but what about shared content across other species. Taxonomic labelling issues are also not fully addressed (e.g. mislabelling, or taxa which share a large amount of genetic material).

We thank the reviewer for raising this point. Given Seqwin estimates specificity based on the non-target genomes and it treats all non-targets equally, it is expected that they only include close neighbors (and optionally well-curated / high-quality ones). Although Seqwin is flexible,

including a large number of distantly related / irrelevant non-targets would significantly bias specificity estimation.

In practice and also in our current pipeline, we BLAST check the output signatures against the whole core_nt BLAST database (consists of almost everything on NCBI), and evaluate the number of hits outside the target genus (targets + neighbors).

Taxonomic mislabelling is hard to address by Seqwin itself. In a future update we will try to weight non-target genomes with Jaccard similarity (also mentioned by Reviewer 4), and this should make specificity more focused on the highly-similar non-targets.

We added these points in the second paragraph of Discussion:

> Seqwin estimates specificity from the provided non-target genomes and considers all non-targets equally. Consequently, specificity is best estimated when the non-target set is restricted to close phylogenetic neighbors, ideally using well-curated and high-quality genomes.
>
> Although Seqwin is flexible and tolerates some noise in the non-target set, including a large number of very distantly related non-target genomes can bias specificity estimation. In practice, it is suggested to perform an additional screening of candidate signatures against a comprehensive background and examine hits outside the intended search space.
>
> Taxonomic mislabelling in public databases is another challenge that Seqwin itself cannot resolve. Using a genome-based taxonomy database such as GTDB may alleviate this issue. A potential solution is to weight non-target genomes by sequence similarity, for example using Jaccard indices, so that specificity estimation is driven more strongly by the most relevant near-neighbor genomes.

- It would be useful to see sensitivity and specificity plots for identification if signatures were used for PCR primers (ignoring biochemical realities of primer design in the first instance). This could be run on a large test database e.g. AllTheBacterian. Are more signatures then better / how many are sufficient? This could be shown for e.g. 1, 2, 3 PCR products. This would more directly relates the computational output to the lab-based protocols this tool is intended to inform.

This is an excellent point. Indeed, in a pathogen surveillance project we've been using Seqwin signatures to design PCR primers internally and we already have a prototype pipeline and some preliminary results. We use varVAMP as our primer design tool - it takes an MSA of the design region as input and can be seamlessly coupled with Seqwin. The general steps of our pipeline are listed below.

1. Extract signatures with Seqwin.
2. For each signature, generate the MSA of the signature regions in all target genomes.

3. Input the target MSA to varVAMP (it does not support an MSA of non-targets).
4. Evaluate the sensitivity and specificity of each assay.

We designed qPCR/ddPCR assays for *M. tuberculosis*, which is also one of the pathogens in our benchmarking datasets. The pipeline was run on Feb. 17, 2026 and 8,570 genomes were downloaded from NCBI, so the output signatures are slightly different from our benchmarking results (done in August 2025). Seqwin found 251 signatures and 169 of them had at least one varVAMP assay. Below is a scatter plot of the sensitivity and specificity of each assay.

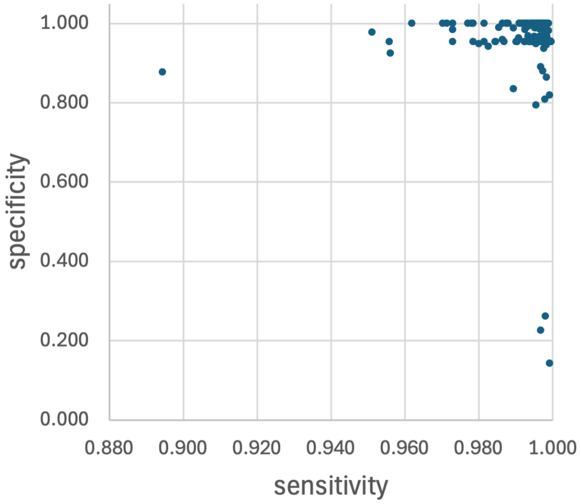

Note that sensitivity and specificity are in silico estimations. An assay is considered sensitive to a target genome if all of the three oligos (2 primers and 1 probe) are matched perfectly. An assay is considered specific to a non-target genome if none of the three oligos is matched perfectly.

However, we respectfully feel rigorous wet lab validation is outside of the scope of this study and we leave as future work.

Other comments:
- "Tens of thousands of microbial genomes are now available" -- millions, arguably tens of millions.

We changed it to:

"Tens of thousands of microbial genomes are now available for a single species".

- Could the tool be applied more broadly, e.g. to diversity within species? Do the authors have any thoughts on applying at the genus level or subspecies levels?

Yes. In fact, the *S. enterica* dataset in the paper is an example of identifying signatures on the subspecies level (using *S. enterica* subsp. *enterica* as target species). Internally, we've been

applying Seqwin on antimicrobial resistant / susceptible strains of the same species (e.g., *Enterococcus faecium*).

There aren't technical limitations of applying the tool to the genus level or above, since Seqwin can take arbitrary sets of target and non-target genomes. However, the number of output signatures could be limited for higher taxonomic levels, due to lower sequence similarity within the target group.

- The difference between k-mers (good for bioinformatic methods in silico typing) and sequences which are good for PCR/WGS targeted is interesting, but not fully explored.

We agree with the reviewer. A good assay target should not only be sensitive and specific, but also exhibit other properties, such as moderate GC content, no complex secondary structures, etc. Seqwin solves the sensitivity and specificity problem, and downstream tools are required to further scrutinize signatures for use as PCR primers. More details can be found in our response to the second major comment from Reviewer 1.

We add the following text to Discussion:

> Although wet-lab validation was not performed in this study (planned as future work), we envision Seqwin being coupled with established primer and probe design software, such as varVAMP, Olivar and PrimalScheme.

- Did the authors consider signatures which are less sensitive/specific, but when weighted (e.g. with ML) produce good results?

This is similar to the question above. There are definitely other metrics to evaluate signatures / genomic targets, and we are considering a ML model to predict the quality of a PCR primer.

- Completeness/metagenome applicability is mentioned at the start, but not demonstrated adequately in the results.

In the Results section, we mentioned "Genomes from all assembly quality levels were included", which means incomplete genomes and metagenome-assembled genomes are included. We included genome completeness in Supplementary Tables, and mentioned in the footnote of Table 2:

> Percentage of complete genomes (assembly level at or above chromosome) in each dataset: 7.43% (*C. difficile*), 12.03% (*M. tuberculosis*), 13.73% (*S. enterica*).

- "For each sequence in a single genome, a set of minimizers is chosen as a subset of all k-mers in the sequence, representing a compressed sketch of the sequence." Didn't understand this. I assume the authors mean the minimizer set is a way of choosing a subset, not that a subset of the minimizers is then taken.

The reviewer’s understanding is correct. We improved this sentence to make it more clear:

> For each sequence in a genome, a set of minimizers is sampled from its k-mers to form the minimizer sketch of that sequence.

---------------------- REVIEW 2 --------------------

SUBMISSION: 402
TITLE: Seqwin: Ultrafast identification of signature sequences in microbial genomes

----------- Overall evaluation -----------
A microbial signature sequence is a DNA sequence that can be used to identify a target microbial group. That is, the sequence should be present in the target genomes and absent in the non-target genomes. The authors present Seqwin, a new method based on minimizer graphs for finding signature sequences given a large database of microbial genomes. Unlike previous tools, Seqwin scales to thousands of genomes and produces more high quality signature sequences than previous methods.

The problem seems relevant, the paper is clearly written, and the experiments look very promising.

I only have one minor comment:
On the dataset published with Fur, only three of the four methods are executed. Why was Neptune left out?

We thank the reviewer for noticing this omission. We have now included the Neptune results for the Fur dataset and updated Table S1, Figures S2 and S3, and the corresponding text in the Results:

> We first used the dataset published with Fur as input genomes for the four tools (Seqwin, Fur, Unikseq and Neptune).

---------------------- REVIEW 3 --------------------

SUBMISSION: 402
TITLE: Seqwin: Ultrafast identification of signature sequences in microbial genomes

----------- Overall evaluation -----------
This manuscript presents seqwin, a method to detect microbial genomic signatures. It uses an undirected graph of minimizers and some ad-hoc graph graph traversals procedures to find the signatures. The manuscript is easy to read because the progression of concepts is clear for

specialists. Results appear to be reasonable/good, especially in terms of computational performance. It is challenging to compare the different tools, because of the multi-dimensionality of results: number of signatures, their lengths, conservation in targets, divergence in non-targets. Seqwin produces neither the largest number of signatures, nor the longest ones on average, but they do appear to be more conserved and more divergent to non-targets on average. Thus in that respect, the tool appears worthy. The methodological contributions are rather straightforward but effective.

Major remarks:
* It is misleading to say that tau_v is automatically computed by the program, because its automatic determination depends on a parameter alpha_v that is asked to be set by the user - or set to 0.5 by default, which is unclear how this value was chosen. A relation question is also, when is it appropriate to let the user determine tau_v*

The reviewer is correct, this is indeed not strictly “automatically computed”. However, the nature of alpha_v is actually “how sensitive and specific do you want your signatures to be?”, and this question can only be answered by the user. Unlike some other tools, Seqwin does not focus on signatures with 100% sensitivity and specificity, so this question has to be answered. We set alpha_v as 0.5 because we assume that most users want a balanced output - a decent amount of signatures with decent sensitivity and specificity.

We emphasized this in the corresponding Methods section:

> In practice, alpha_v defaults to 0.5 and can be tuned by the user to trade off the number of output signatures against their quality (*e.g.*, increase alpha_v when more signatures are needed).

* In Figure 2, many of the signatures have low divergence to non-targets. This appears to be true across all tools. Shouldn’t high-quality signatures display high divergence to non-targets? This point should be discussed.

The reviewer is correct - high-quality signatures usually have high divergence, and the output signatures are not all considered high-quality. This is because signatures are not found with alignment-based methods. For Seqwin and many other tools, they use k-mers to find signatures and they cannot accurately estimate signature specificity. Thus, we need to rely on tools like BLAST to evaluate specificity.

Seqwin sorts the output signatures by the sum of conservation and divergence, and the high-quality ones are placed at the top (Supplementary Note 2).

* A comparison with NAUniSeq is needed, even though the authors noted that it uses all of RefSeq. Still, that method detects genomic signatures, therefore it is legitimate to report its real-world performance.

We thank the reviewer for suggesting a comparison with NAUniSeq. We carefully examined both their codebase and their paper. However, we concluded that including NAUniSeq in a benchmark would be difficult to do in a reliable and reproducible manner, and that the comparison would likely be of limited interpretive value for readers for the following reasons.

1. The current GitHub repository for NAUniSeq (https://github.com/Gulshan-gaur/NAUniSeq) is incomplete: most of the source code is no longer available, and the installation and usage instructions are not clear enough for us to reproduce the method with confidence. In addition, the download link for the test data is no longer active, and the "test_data" folder has also been removed from the repository (returns a "404 FILE NOT FOUND" error).

2. Based on the description in the paper (https://academic.oup.com/bib/article/25/6/bbae545/7832362), their method evaluates exact matches of long k-mers (k=100): for each 100-mer in the target genomes, check if an exact match exists in the non-target genomes. Qualified 100-mers are directly output as signatures, shown in Table 2 and 3 of the paper. The other k-mer-based methods (e.g., Unikseq and Neptune) combine qualified k-mers into longer signatures, and they use smaller and more common k values (e.g., 20-30).

In summary, we concluded that NAUniSeq follows a simpler approach than the other k-mer-based methods, and the currently available code and documentation were not sufficiently complete to enable a reliable and reproducible evaluation. We updated our review of k-mer-based methods in Introduction, and the reason why NAUniSeq was not included in Results:

> Methods such as Neptune, KmerGO, KEC and Unikseq use alignment-free k-mer filtering and subsequently combine k-mers into longer signatures through sequence assembly or similar strategies. However, they all require substantial memory to store k-mers from all input genomes.
>
> NAUniSeq is a recent k-mer-based method that automates the selection of target and non-target taxa, reporting identified k-mers directly as signatures without further extension. As a result, it typically requires a larger k (e.g., 100 bp), and all output signatures are fixed at that length.
> …
> Note, NAUniSeq was not included in the benchmark given we were unable to successfully run it on our evaluation dataset due to missing files that caused installation errors.

Minor remarks:

* How widespread is the term "signature sequences"? In the Introduction, only one reference to a 2003 review is given, please consider adding more references to that definition and/or explain the status of this term with respect to the microarrays/pre-NGS/NGS eras. For instance, some of

the references later given ([7] through [13]) instead use the term “oligonucleotide signature” in their title. Later in the Introduction, you also use the term “genomic signature”, and “marker discovery” (for NAUniSeq), so for the sake of consistency, make it explicit that they refer (or not) to the same task.

We apologize for the confusion. We cited the 2003 review (Slezak et al.) because to our knowledge it was the first paper that defined the modern “DNA signature” concept, quote: “Detection signatures need to be both conserved over all target strains and unique compared to all non-target genomes.”

The terms “signature sequence”, “oligonucleotide signature”, “genomic signature”, etc., all refer to the same concept, and the tools cited in Introduction all try to solve the “signature discovery” problem. Some of them didn’t use the word “signature” in their paper, but they all take inputs from a target group and a non-target group, which is the core of the signature concept.

Going into the NGS era, a slight change was made in the definition of signature. A signature is no longer required to be 100% conserved in all target genomes, due to the increasing size and diversity of public databases.

We emphasized these points in Introduction:

> DNA signature sequences were originally defined to be sequences both 100% conserved over all target genomes and unique compared to all non-target genomes (cite Slezak, 2003). However, over time the definition has been relaxed in signature detection tools and is now more commonly defined as a sequence that is highly sensitive, (largely present in most target genomes) and highly specific (largely absent in or divergent to closely related non-target genomes).
> …
> While some of these tools do not explicitly use the term “signature”, they computationally address the same problem using target and non-target genome groups as input.

We also changed “marker discovery” to “signature discovery” for NAUniSeq.

* Also in the early Introduction, the concept of “nearest-neighbor thermodynamic filtering” is obscure.

We apologize for not making this clear. By “nearest-neighbor thermodynamic filtering” we meant filtering candidate oligos based on thermodynamic metrics (e.g., melting temperature and delta G) calculated with a nearest-neighbor model (e.g., SantaLucia, 1998). However, this is still inaccurate because YODA and ProDesign also use other filtering criteria. Therefore, we changed the statement to this:

> Approaches such as YODA and ProDesign use exhaustive searches combined with various filtering criteria.

* The sentence “pragmatic way of estimating tau_v” can be misleading, given that there is no ground truth for what tau_v should be.

The reviewer is right that in practice there is no “true value” or “the best value” of tau_v. But we used “estimating” here because we proposed an intuitive way to calculate tau_v, defined in Equation (4), and its value can be accurately calculated if you count all k-mers in all genomes. Since listing all k-mers is very expensive, we use either MinHash sketches or minimizer sketches to estimate this value.

We rewrote this sentence to make it more clear:

> Consequently, this provides a practical solution for estimating τ_v defined in Equation (4), particularly when processing large datasets where calculating pairwise Jaccard indices is computationally prohibitive.

* The use of POA (partial order alignment) to construct the final minimizer order appears natural, but is not discussed. Perhaps worth considering for an extension of the method.

We thank the reviewer for suggesting the use of POA, and we think it will be a promising extension to explore. Currently we use the most predominant minimizer ordering as the “representative”, to make sure that the output signature comes from one of the target genomes. Alternatively, we can make a POA graph of all minimizer orderings, and get the “consensus ordering”. We can compare this consensus to the representative ordering, and see if they are the same. We can also check if the consensus exists in one of the genomes. These could increase our confidence in the output signatures.

---------------------- REVIEW 4 --------------------

SUBMISSION: 402
TITLE: Seqwin: Ultrafast identification of signature sequences in microbial genomes

----------- Overall evaluation -----------
Wang et al. describe Seqwin, a new computational method for deriving genomic signatures that discriminate from on-target and off-target groupings of genomes. The main technical advance is to use minimizer graphs to collapse genomes into a pseudo-assembly graph, and then find components that are consistently enriched in on-target genomes and depleted in off-target genomes.

The technical idea is nice, and the problem is important to some microbiologists who are interested in PCR-based discrimination of microbes. The paper is quite easy to read, and all the technical details check out. Most technical steps are extensions of known techniques (e.g. extending pan-minimizer graphs from ntJoin or other adjacency-based marker approaches like

panaroo) with some new ideas, and also using well thought-out heuristics. But it appears to be effective. The benchmarking also seems reasonable.

I only have a few minor comments.

- dashed lines and lightly-colored nodes could be explicitly labelled in Fig. 1D for clarity

We defined them more clearly in the caption:

> … light blue, light green and dashed lines are minimizers and edges not included in this subgraph.

- Is there any justification for using L2 distance over other distance metrics? I only bring this up because the authors seem to emphasize the L2 norm in their method description.

This is a good question brought up by the reviewer. The reason we chose the L2 norm to calculate penalty is its geometric interpretation. If you make a scatter plot of all minimizers, where x-axis is sensitivity and y-axis is specificity, the penalty threshold will be a quarter-circle arc at the upper right corner of the plot - minimizers within this arc are considered both sensitive and specific. We think this is a natural way of selecting the minimizers that form signatures.

- I worry a bit about the use of expectation in Equation 4. What could happen is that a few of the off-target genomes are really similar (~> 99%) to the target genomes, but all other off-target genomes are dissimilar. The expected number of shared k-mers is low, so the resulting threshold is low. But you should really be focusing on those "highly-similar" off-target genomes. I'd encourage the authors to think of using the distribution of Jaccard similarity, rather than the point estimate of a mean, to automatic tune the threshold.

The reviewer's concern is valid. In the algorithm, we assume all non-target genomes are equal, and a few highly similar non-target genomes should not significantly affect specificity. However, this is under the assumption that the user only selects the non-targets that they care the most, instead of including distantly related, irrelevant genomes. In future updates of Seqwin, we could try to weight non-target genomes by Jaccard similarity, and use these weights to calculate "minimizer presence in non-targets".

We added these points in the second paragraph of Discussion. Specifically,

> A potential solution is to weight non-target genomes by sequence similarity, for example using Jaccard indices, so that specificity estimation is driven more strongly by the most relevant near-neighbor genomes.

- "those overlapped with confirmed MGEs had relatively lower divergence (Figure 3)." please quantify

First, we apologize that this sentence is not clearly written. We rewrote it and also calculated the median divergence of signatures overlapping with insertion sequences:

> In all three experiment settings, less than 10% of the signatures overlapped with predicted MGEs (compositional outliers and insertion sequences). Moreover, only *M. tuberculosis* had signatures overlapping with insertion sequences (Figure 3), and their median divergence is 0.021, which is lower than the median divergence of all *M. tuberculosis* signatures (Table 2).

- "under three pathogenic taxa and their neighboring taxa:" I had to look at the Supp. tables to confirm what "neighboring taxa" meant -- clearly distinct taxa that are similar species/genera. Authors should be very explicitly how they chose neighboring taxa, sicne this is critical for benchmarks.

We apologize for not making this clear. We added this sentence after the one mentioned by the reviewer:

> Here, “neighboring taxa” are those that diverge from a common ancestral node into separate sister lineages. For example, as the only species in the genus Clostridioides, *C. difficile*’s neighbors encompass all other genera under the shared family, *Peptostreptococcaceae*.

----------------------- REVIEW 5 ---------------------

SUBMISSION: 402
TITLE: Seqwin: Ultrafast identification of signature sequences in microbial genomes

----------- Overall evaluation -----------
The authors present Seqwin, a novel method to identify signature sequences, genetic regions with high occurrence in a desired target group and absent within non-target genomes. Seqwin identifies non-exact target sequences across tens of thousands of genomes applicable for downstream polymerase chain reaction (PCR) diagnostics. To identify signature sequences, the authors propose a weighted pan-genome minimizer graph-based approach created from the combined undirected minimizer graph for each genome. Disjoint low-penalty subgraphs are greedily selected from the scored and filtered pan-genome minimizer graph. Then representative sequences from each subgraph are selected based on the maximal consecutive occurrence of the subgraph’s minimzers across the target genomes. The authors evaluate Seqwin against three existing tools (Fur, Unikseq, and Neptune) and demonstrate that Seqwin identifies valid candidate signatures with high sensitivity and specificity (as evaluated using BLA ST), in less or comparable runtime and memory than existing tools. The authors acknowledge some flaws and potential improvements on Seqwin, but, as is, Seqwin already presents a practical advancement for large databases that is easily downloadable and usable through bioconda. Overall, the manuscript is clear and very well written and proposes a novel, efficient

approach to identifying candidate signature sequences for PCR diagnostics. I have a few comments split into major and minor concerns:

Major concerns/comments:
* Are the signature sequences output from Seqwin similar to those output by Fur, Unikseq, and Neptune? Do these tools identify good signature sequences that are missed by Seqwin?

This is a great point raised by the reviewer. To showcase this, we BLAST aligned Seqwin signatures against signatures from other tools. We chose BLAST because each tool might use a different target genome to extract the actual sequence of a signature, so it would be hard to compare signature similarities based on sequence coordinates. To ensure a valid estimation, we chose the megablast algorithm to find high-confidence alignments between signatures. We further filtered out alignments less than 99% nucleotide identity.

For each Seqwin signature with a BLAST hit against signatures from another tool, we report "alignment percentage" = "length of alignment" / "length of Seqwin signature". For example, a Seqwin signature has length of 500 bp, and 300 bp of it is aligned to a Fur signature. Then its alignment percentage is 60%.

Results are shown in the figure below (best interpreted jointly with Figure 2 in the manuscript). Each data point represents a Seqwin signature aligned to a signature from another tool, and y-axis is alignment percentage. The title of each subplot indicates "# aligned signatures / # total Seqwin signatures".

In some settings, Unikseq generated a large number of signatures (see Figure 2), and almost every Seqwin signature aligned with a Unikseq signature. For Fur and Neptune, there were fewer shared signatures. There were some signatures that were missed by Seqwin (e.g., Unikseq had many low-conservation ones), that require further investigation. However, since most Seqwin signatures were determined to be good signatures, Fur and Neptune missed many high-quality signatures.

For *S. enterica*, very few Seqwin signatures aligned with signatures from other tools as they struggled to output high-quality signatures in this setting (Figure 2).

In summary, there are scenarios where Seqwin misses (good) signatures identified by other tools. However, Seqwin consistently finds good signatures even in challenging datasets, and most of them are missed by the other tools.

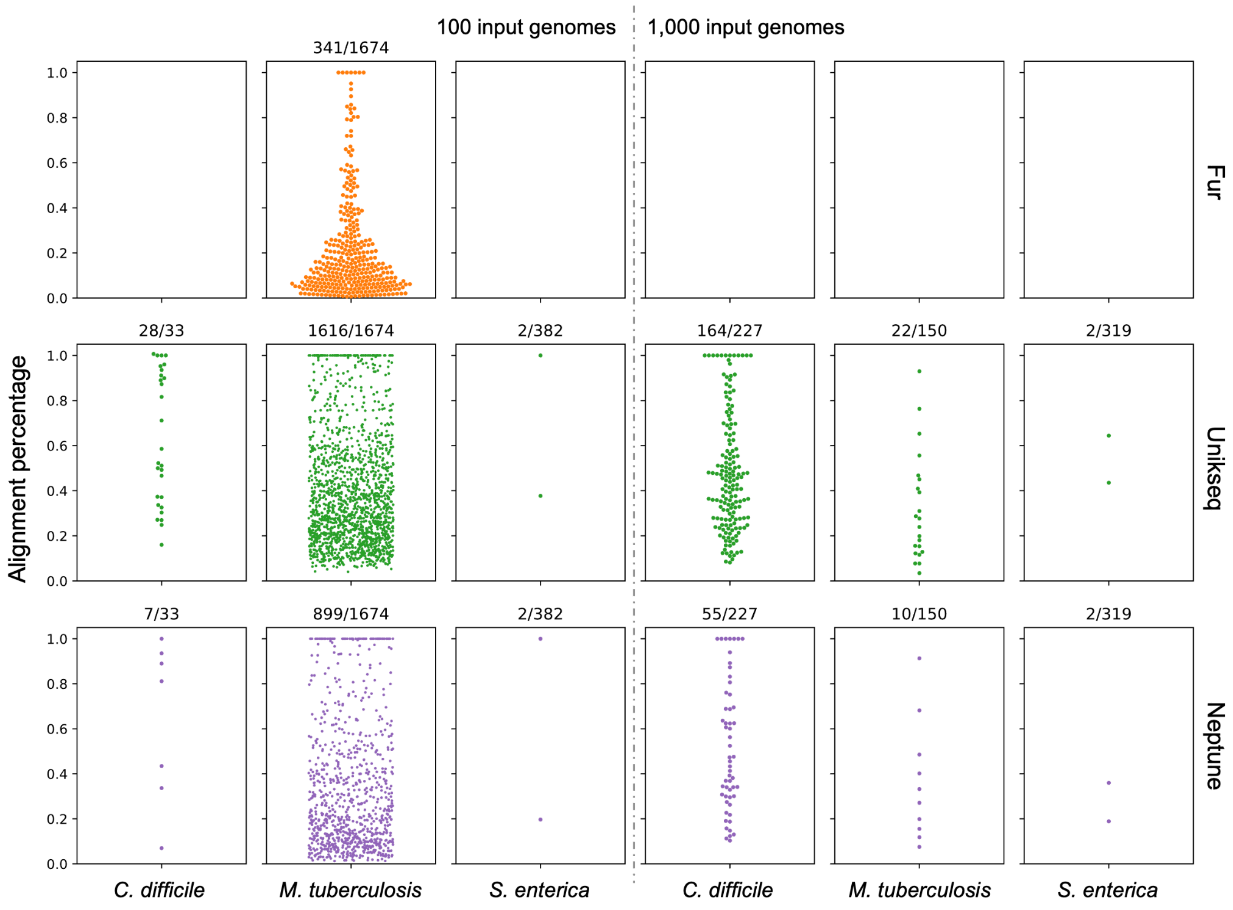

* In choosing a representative sequence for each low-penalty subgraph, are the representative sequences constrained to always start and end at a minimizer? Can an extension or shift of this sequence yield a better signature?

Yes, representative sequences always start and end at minimizers, including the minimizer sequences themselves. The algorithm also tries to extend the low-penalty subgraph during BFS extension, as long as the average node penalty stays below the threshold. Thus, further extension or shifting of the subgraph would probably decrease the sensitivity and specificity of the signature.

Minor concerns/comments:
* Italicize scientific names (for example, “C. difficile, M. tuberculosis and S. enterica” in the Abstract). Also recommend the Oxford comma here

Thank you for pointing this out; we tried to italicize those names but it didn’t work in the Abstract text box entry on the paper submission website. We will get it fixed, and for now, think it’s probably best to leave this issue to the editorial and publishing team. The Oxford comma was added.

* How often does Seqwin miss good candidates? Include an experiment to evaluate this, such as calculating divergence and conservation of a ~200bp scanning window (perhaps just evaluating on a genome from group A of the 33 E. coli as your target against the other non-A groups)

This is an excellent follow-up question of the first question of the reviewer. We did not include the specific experiment suggested here because it would require millions of BLAST searches and was beyond the scope of the revision. However, we can comment on a few simpler cases.

1. A SNP is presented in all targets, and absent in all non-targets.
In this case, Seqwin will likely miss this signature under default settings (k=21, w=200), since only ~1% of all k-mers are selected as minimizers.

2. A large insertion is presented in all targets, and absent in all non-targets.
If the length of the insertion is ≥ k + w -1, at least one of its k-mers will be selected as a minimizer. Then this minimizer will be picked up by Seqwin since its penalty is 0 and is definitely below the threshold. However, by default Seqwin requires at least 3 minimizers to support a signature. In that case, the length of the insertion should be ≥ k + 3w -1 to make sure it is included as a signature. In practice, signatures much shorter than that can be selected since the expected span of 3 minimizers is w + 1 (discussed in Methods).

In practice, the definition of "good candidates" depends on the penalty threshold. If the threshold is set too low, you might miss candidates with lower but still decent sensitivity and specificity.

"Combinatorial signatures" are another type of signatures that Seqwin cannot identify, and we discussed this in Discussion:

> Seqwin is designed to identify single genomic regions that distinguish the target group from the non-target group, rather than combinations of multiple regions that are informative only when considered together. As a result, Seqwin may miss combinatorial signatures in which no individual region is sufficiently conserved across the full target set, even though the union of those regions could still distinguish targets from non-targets.
>
> This limitation is relevant when designing assays for antimicrobial-resistant (AMR) strains of a pathogen, as AMR can depend on combinations of mutations or genes across multiple loci rather than on a single diagnostic region. Support for identifying combinatorial signatures is under development and is planned for a future Seqwin update.

* During PCR diagnosis of a novel outbreak, there may only be a few assemblies of the target species. How does Seqwin perform when there may be only 1 or 2 available target genomes?

Seqwin does work when there are only 1 or 2 target genome assemblies. However, in that case users should provide a penalty threshold instead of relying on the automatically calculated one (Equation 4), which is based on k-mer statistics and would only make sense when there are sufficient target genomes. When there are only 1 or 2 target genomes, the penalty threshold is only determined by expected k-mer presence in non-targets, since k-mer presence in targets could only be 0%, 50% or 100%. For example, users may set the penalty threshold to 0.1, and Seqwin will find k-mers present in all target genomes and in less than 10% of non-target genomes.
